# Changes in speech, language and swallowing services during the Covid-19 pandemic: The perspective of speech-language pathologists in Saudi Arabia

**DOI:** 10.1371/journal.pone.0262498

**Published:** 2022-01-18

**Authors:** Nisreen N. Al Awaji, Alanoud A. AlMudaiheem, Eman M. Mortada

**Affiliations:** 1 Department of Health Communication Sciences, College of Health and Rehabilitation Sciences, Princess Nourah Bint Abdulrahman University, Riyadh, Saudi Arabia; 2 King Abdullah Specialized Children Hospital-King Abdulaziz Medical City, Riyadh, Saudi Arabia; 3 Health Sciences Department, College of Health and Rehabilitation Sciences, Princess Nourah Bint Abdulrahman University, Riyadh, Saudi Arabia; Kasturba Medical College Mangalore / Manipal Academy of Higher Education, INDIA

## Abstract

**Purpose:**

The study aimed to investigate changes in the role of speech-language pathologists (SLPs) during the COVID-19 pandemic in Saudi Arabia. It also assessed the SLPs’ perceptions of delivering services using telehealth as a part of their everyday clinical practice before and during the COVID-19 pandemic.

**Method:**

SLPs in Saudi Arabia were invited to complete a web-based survey covering questions related to changes to the role of SLPs during the COVID-19 pandemic, changes in the ways speech services are delivered; and the benefits and barriers of using telehealth in clinical practice as identified by SLPs.

**Results:**

Ninety-one SLPs completed the survey. About 94% of the respondents experienced changes in their role as a response to the COVID-19 pandemic. The nature of changes they had experienced including decreased work time, providing support and counseling to patients or caregivers using the telephone, providing assessment and therapy using telehealth, and working with a limited number of cases. Ninety-three percent of the respondents who have used telehealth started to use it only during the pandemic. Mostly seen caseloads were pediatric speech and language disorders. Further, 96% of respondents used telehealth for counseling, 69% for rehabilitation or treatment, 63% for screening, 56% for evaluation or assessment, 48% for a referral to other professional services, and 46% for differential diagnosis. About 70% of the SLPs showed interest to continue using telehealth in the future. Several benefits were identified to using telehealth, including accessibility, cost efficiency, and the ability to engage patients with their families in therapy sessions. On the other hand, barriers to using telehealth included internet and technical issues, lack of direct communication, and difficulty controlling the therapy setting.

**Conclusions:**

The study has shown that SLPs in Saudi Arabia have experienced changes during the COVID-19 outbreak. The survey responses also indicate that the SLPs are adopting telehealth applications at an accelerated pace as a result of the pandemic.

## Introduction

In late 2019, a new strain of coronavirus (COVID-19), originating in Wuhan, China, began spreading rapidly and has become a major challenge for countries all around the world. In February 2020, COVID-19 had been identified in 34 countries, with more than 80,000 confirmed cases and 2,700 deaths around the world [1], leading WHO to categorize the disease, caused by severe acute respiratory syndrome coronavirus 2 (SARS-CoV-2), as a global pandemic [2].

In response to the COVID-19 pandemic, many countries have applied measures and restrictions intended to limit the spread of the pandemic. These include travel restrictions, social distancing, self-isolation, work-from-home, closures of educational institutions, gyms, and places suited to large gatherings, and ceasing some services, including rehabilitation [3]. A growing number of courses, programs, workshops, conferences, and other activities have shifted from traditional modes (face-to-face) to the online delivery mode.

Further efforts to combat the impact of the COVID-19 pandemic involve the prioritization of some health services with the result that non-urgent appointments and outpatient clinics were canceled or rescheduled. These include therapeutic services (e.g., speech-language pathology (SLP) and physiotherapy), non-emergency dental treatment, laboratory services, pharmacy, non-urgent elective surgery, diagnostic imaging, and non-urgent general radiology [4–6]. As a result, the relationship between healthcare providers and consumers has suffered an interruption due to social distancing. Consumers who had been undertaking regular visits and systematic rehabilitation in speech therapy clinics were no longer able to receive face-to-face care due to the COVID-19 precaution measures. However, in other clinics, services were temporarily discontinued, and this was evident in a recent study conducted in Saudi Arabia where most of the caregivers reported suspension of speech therapy sessions during the COVID-19 pandemic [6]. On the other hand, many SLPs have started providing their services via telehealth during the pandemic to avoid the risk of transmission [7–9].

Telehealth is the utilization of telecommunications in health and medical services using tools such as telephone and video conferencing, mobile devices, and e-mails [10, 11]. It is one of the service-delivery options within the scope of practice of SLPs recognized by the American Speech-Language-Hearing Association (ASHA) and Australia Speech Pathology [12].

Several studies around the world have investigated the perception and use of telehealth among SLPs. One such study was conducted in the USA and used a survey developed by ASHA [13] to assess the use of telehealth as part of service delivery by both audiologists and SLPs. Of the 825 SLPs who responded to the survey conducted in 2002, only 9% were using telehealth for service delivery; however, 47% SLPs who were non-users showed an interest in the use of telehealth services. Nearly 70% of SLP respondents used telehealth to provide counseling and follow-up services, whereas a few percent used telehealth for treatment (23%) and screening (18%). The service was used in different clinical areas, including cognitive communication and motor speech disorders.

The other survey was conducted more recently and recorded the responses of 476 SLPs [14]. Of those who responded, 64% (n = 307/569) were using telehealth, 57% (n = 175/307) of whom had already been using it for more than three years. Almost all the SLP respondents used telehealth primarily for treatment and, to a lesser degree, for assessment. Telehealth services were used in a variety of clinical areas, including language disorders articulation, and phonological disorders.

A further study was conducted in Australia to determine the clinical use of telehealth among SLPs who were already using telehealth in their practice. Fifty-seven individuals responded to the survey, which reported that 80.8% had been using telehealth for more than six years. Most respondents were using telehealth for treatment services and, most often, in expressive language and fluency among the pediatric population. For the adult population, telehealth services were more commonly used in therapies related to fluency and dysarthria. Respondents also reported their experiences using telehealth and provided generally positive feedback. In India, a survey was also conducted to investigate the perception and application of telehealth among SLPs. As indicated by the 205 SLP respondents, there was a shortage of clinicians in India. Telehealth may be a solution for such an issue; however, if it is to be adopted nationwide, some requirements must be met, including the improvement of infrastructure, the training of clinicians, and the creation of clear telehealth policies [15, 16].

Existing studies recognize the critical role played by telehealth in overcoming physical barriers by providing patients and caregivers with access to health care, as well as its ability to limit the discontinuity of patient care, especially for outpatient clinics. Furthermore, it enables clinicians to treat more patients and bridge mobility and distance gaps for elderly, disabled, and rural patients [17, 18]. The COVID-19 pandemic has highlighted these benefits even further: under government recommendations and, in some cases, orders to stay-at-home and social distance, telehealth healthcare systems can play a major role in sustaining access to health care and reducing the risk of patient harm caused by these emergency health measures [19, 20].

Adoption of telehealth by SLPs has increased significantly in different countries during the COVID-19 pandemic which has been provided as an alternative service without compromising individual safety. Most of the SLPs have exhibited a positive attitude toward the use of telehealth in the future which proved a fast response to the changes brought on by the COVID-19 pandemic [21, 22]. The use of telehealth was judged to be adequate for different clinical practices (e.g., speech and language disorders, assessment and therapeutic interventions, and counseling), but less for other practices (e.g. swallowing and feeding disorders, orofacial myofunctional disorders, childhood apraxia of speech) [22].

In Saudi Arabia, strong signs have been observed of speech-language pathologists responding rapidly and creatively to the changes by delivering services differently. Therefore, to better understand the impact of COVID-19 on speech therapy provision, a survey was developed to explore the changes in service provision and the perspectives of SLPs on these changes. The research aims for the survey in this study were:

1. To explore how the COVID-19 pandemic caused changes in the role of SLPs in Saudi Arabia and how these changes impacted individual professionals.
2. To assess the perception of SLPs in Saudi Arabia in introducing telehealth as a part of their everyday clinical practice.

## Materials and methods

### Study design and participants

A cross-sectional survey study was carried out in the Kingdom of Saudi Arabia between June and July 2020, during the COVID-19 pandemic. The online survey was distributed through e-mails and different social media platforms to all practicing SLPs in Saudi Arabia.

### Survey

A survey was generated using the Google Forms online survey system and consisted of the following three parts: the first part elicited information from SLPs related to their age, gender, marital status, nationality, city, years of experience, qualification, workplace, and area of practice. The second part included questions to determine how the role of SLPs and the delivery of speech services had changed since the onset of the COVID-19 pandemic. The third part gathered the perspectives of SLPs in Saudi Arabia on the benefits of and barriers to using telehealth in clinical practice.

### Ethical considerations

The study was ethically reviewed and approved by the Institutional Review Boards (IRB) at Princess Nourah Bint Abdulrahman University (IRB log number: 020–0251) in Riyadh, Saudi Arabia. The study information was provided in the first page of the survey description, as was the authors’ contact e-mail. A consent statement and question were also added at the beginning of the survey, with respondents required to check a box to indicate consent and agreement to participate in the study before accessing the survey. Respondents were also assured of the confidentiality of their information and the right to withdraw from the study at any time.

### Statistical analysis of data

Data collected were analyzed using the Statistical Package for the Social Sciences, SPSS (V. 20). Descriptive statistics using frequency and percentage for categorical variables were used for analysis, and results related to the SLPs’ perceived benefits of and barriers to using telehealth were categorized, coded, then analyzed.

## Results

### Respondents demographics

A total of 94 SLPs provided their consent to participate in the study, although three incomplete responses were excluded. Ninety-one speech-language pathologists were included in data analysis. The characteristics of respondents are presented in Table 1. Among the respondents, 87% were female, and 44% were within the age range of 26 to 35 years old. SLPs reported the following years of experience working in the field: 31.9% responded “*less than 2 years*,” 26.4% responded “*more than 10 years*,” 22% responded “*2–5 years*,” and 19.8% responded “*6–10 years*.” Almost two-thirds of the participants possessed a bachelor’s degree (64.8%), 28.6% had master’s degrees, and 6.6% held doctorates. Professional titles were distributed as follows: intern (3%), therapist 1 (41%), therapist 2 (23%), senior therapist (23%), consultant (6.6.%), and others (3%).

**Table 1:** Personal and professional practice-related characteristics of respondent SLPs (n = 91).

Characteristics	Responses	No (%)
**Age:**		
◦ ≤25 years	30	33.0
◦ 26–35	40	44.0
◦ >36	21	23.1
**Gender**:		
◦ Male	12	13.2
◦ Female	79	86.8
**Marital Status:**		
◦ Not Married	52	57.1
◦ Married	39	42.9
**Nationality:**		
◦ Not Saudi	13	14.3
◦ Saudi	78	85.7
**Years of Experience:**		
◦ less than 2 years	29	31.9
◦ 2–5	20	22.0
◦ 6–10	18	19.8
◦ more than 10	24	26.4
**Qualification:**		
◦ Bachelor	59	64.8
◦ Masters	26	28.6
◦ PhD	6	6.6
**Professional Title**		
◦ Intern	3	3.3
◦ Therapist 1	37	40.7
◦ Therapist 2	21	23.1
◦ Senior Therapist	21	23.1
◦ Consultant	6	6.6
◦ Others	3	3.3
**Workplace:**		
◦ Private clinic	18	19.8
◦ Governmental hospital	43	47.3
◦ University hospital	8	8.8
◦ Public school	1	1.1
◦ Private school	12	13.2
◦ Others	9	9.9

Among all respondents, almost half (47.3%) worked in governmental hospitals at the time of the survey, followed by 20% who worked in private clinics. When asked to choose an area or areas of practice, most respondents indicated that they worked with pediatric speech (85.5%) and language (78%), followed by pediatric fluency (56%), adult speech (48%), adult fluency (41%).

### Professional practice during the COVID-19 pandemic

**Fig 1:**
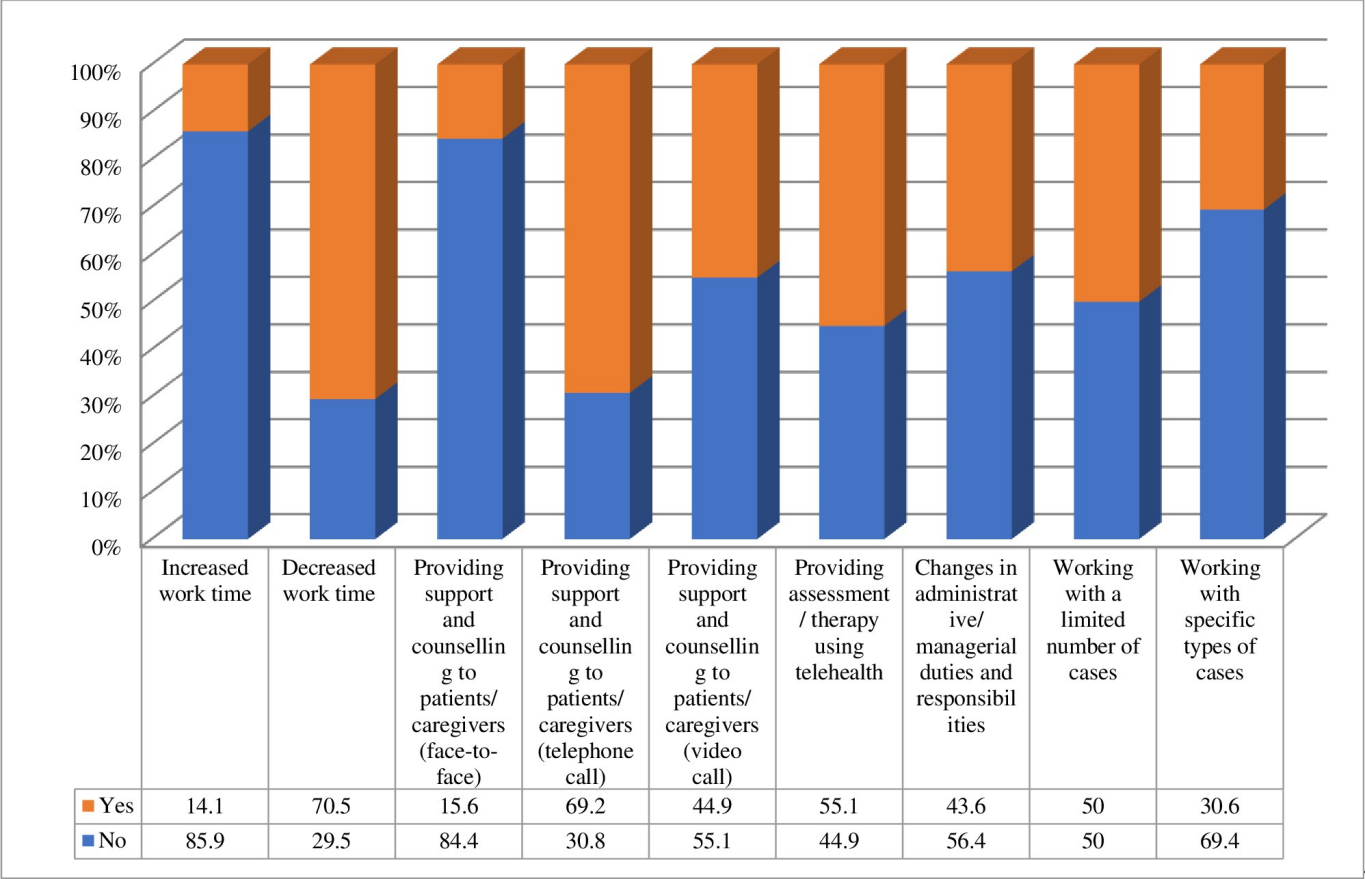
Changes experienced by SLPs during the COVID-19 pandemic (n = 86).

The vast majority of participants (94.5%) said that they experienced changes in their practice and responsibilities during the COVID-19 pandemic. When asked to select from a list of options the nature of changes they had experienced, 70% indicated decreased work time, 69% provided support and counseling to patients or caregivers using the telephone, 55% provided assessment and therapy using telehealth, and 50% worked with a limited number of cases (nature of changes are shown in Fig 1).

### Use of telehealth by SLPs

In response to the question “Have you ever used telehealth in your practice?”, 67% (n = 61/91) of respondents said *yes*, and 80% reported having used telehealth for less than six months. Specifically, 93% reported that they had started to use telehealth during the COVID-19 pandemic (Table 2).

**Table 2:** Use of telehealth by respondent SLPs.

Characteristics		Responses	No (%)
	**Ever used telehealth in practice?**		
	◦ No	30	32.9
	◦ Yes	61	67.1
**SLPs replied yes**	**How long you have been using telehealth?**		
	◦ Less than 6 months	49	80.3
	◦ 6–12 months	7	11.5
	◦ 13–24 months	3	4.9
	◦ More than 2 years	2	3.3
	**Use telehealth during the COVID-19 pandemic lockdown?**		
	◦ No	4	6.8
	◦ Yes	55	93.2
	**Like to continue to use telehealth in the future?**		
	◦ Strongly agree	23	39.0
	◦ Agree	19	32.2
	◦ Uncertain	9	15.3
	◦ Disagree	4	6.8
	◦ Strongly disagree	3	5.1
**SLPs replied no**	**Would you like to use telehealth in the future?**		
	◦ Strongly agree	9	33.3
	◦ Agree	8	29.6
	◦ Uncertain	10	37.0
	◦ Disagree	0	0.0
	◦ Strongly disagree	0	0.0
	**Think telehealth would be an effective method to be practiced by SLP in the future**		
	◦ Strongly agree	6	22.2
	◦ Agree	8	29.6
	◦ Uncertain	7	25.9
	◦ Disagree	3	11.1
	◦ Strongly disagree	3	11.1

Among the respondents who said *yes* to the question, 39% selected “*strongly agree*” to use telehealth in the future, while 32% selected “*agree*.” On the other hand, of the respondents who said *no* to the question, 37% were uncertain whether they wanted to use telehealth in the future, 33% selected “*strongly agree*,” and about 30% selected “*agree*.” Respondents were also asked to indicate whether they thought “telehealth would be an effective method to be practiced in the future”: 22% selected “*strongly agree*,” about 30% selected “*agree*,” and 26% were “*uncertain*.”

**Fig 2:**
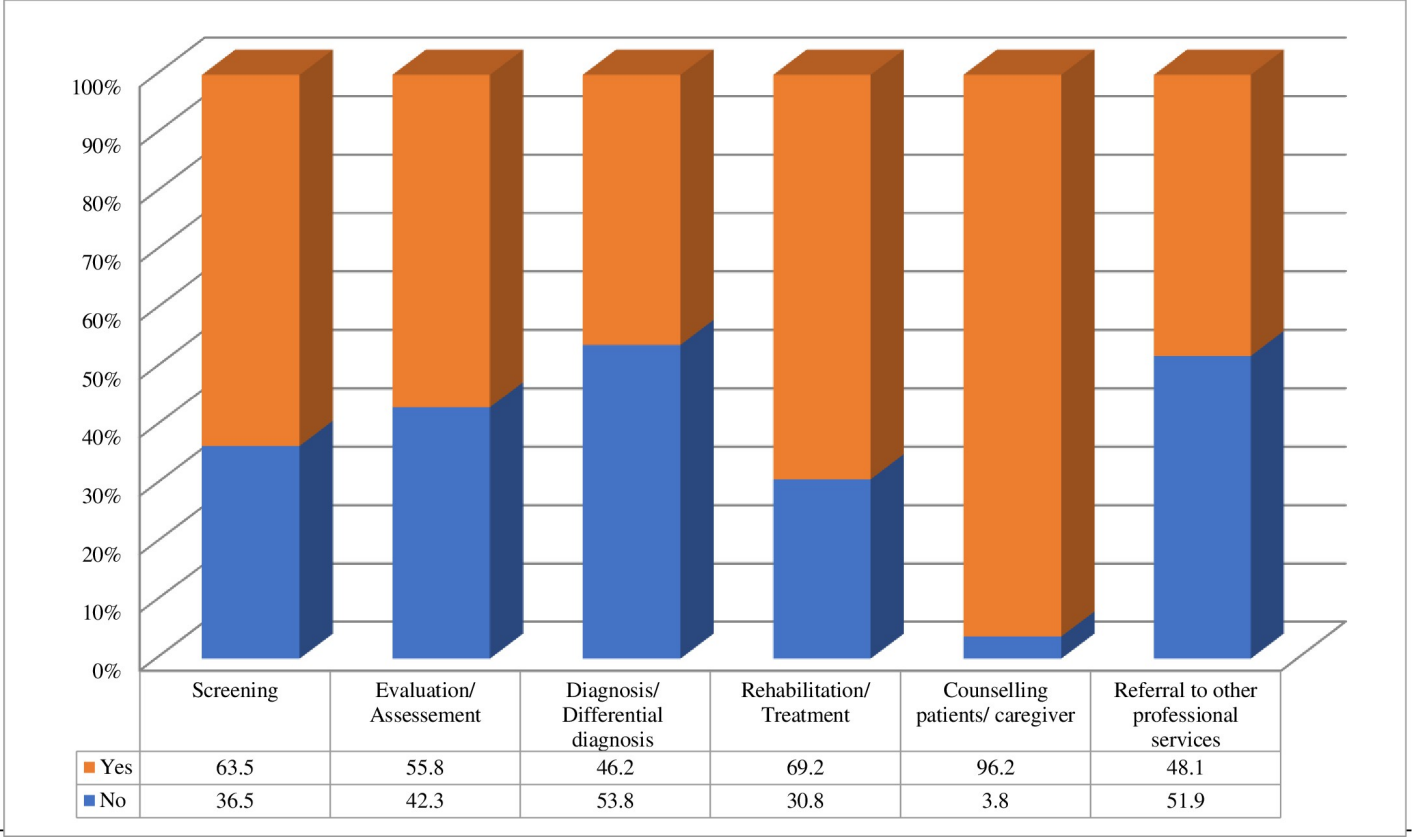
What services did/do you use with your patients via telehealth? (n = 61).

Turning now to the type of service or services used with patients via telehealth, 96% respondents used telehealth for counseling, 69% for rehabilitation or treatment, 63% for screening, 56% for evaluation or assessment, 48% for a referral to other professional services and 46% for differential diagnosis. The survey demonstrated that most of the cases seen by the SLPs via telehealth were pediatric language disorders (80%), followed by pediatric speech disorders (70%), pediatric stuttering (44%), and adult speech disorders (40%) (Fig 2).

**Fig 3:**
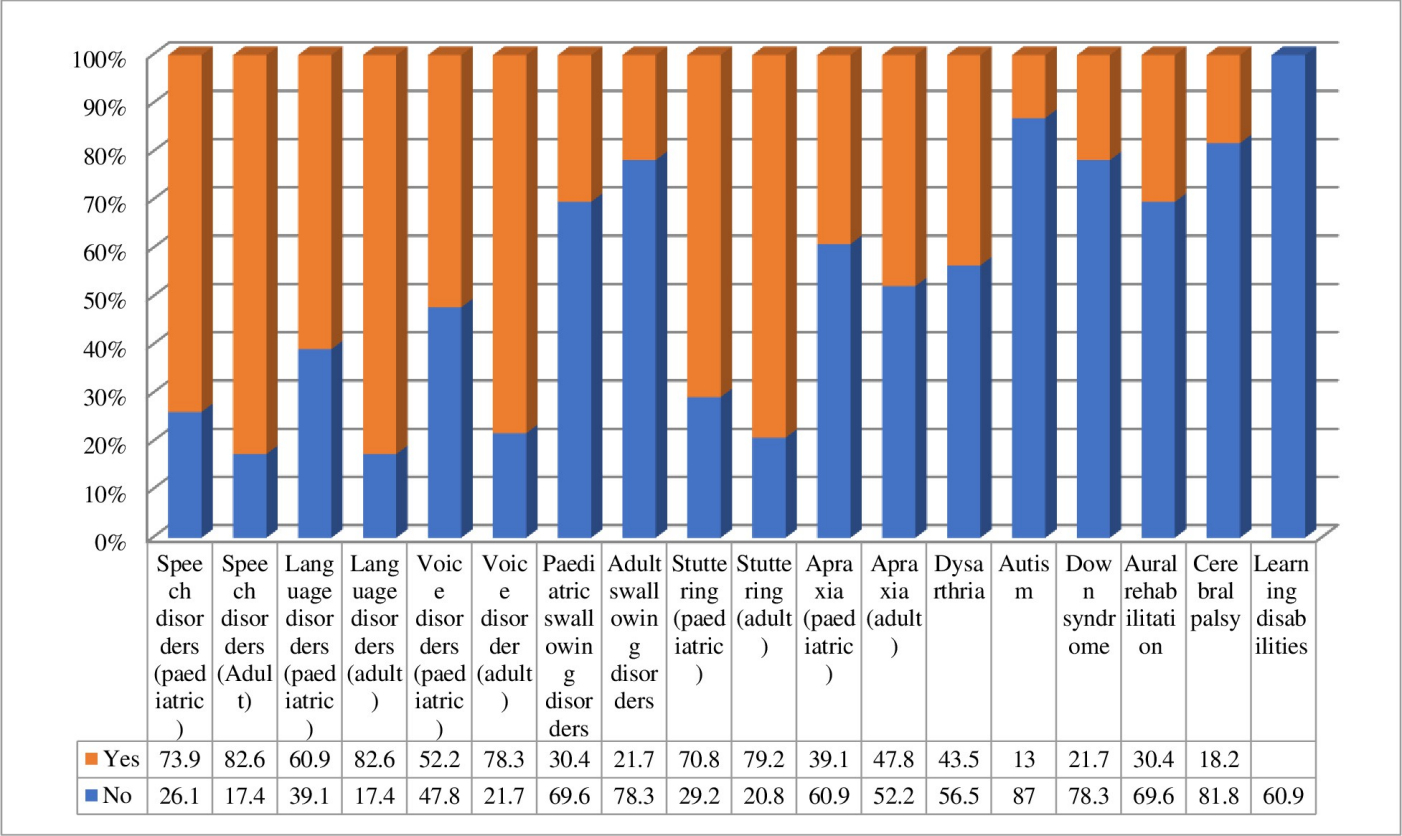
Areas of practice where telehealth can replace conventional speech therapy in the future (n = 30).

In response to the question ‘Which areas do you think telehealth can replace conventional speech therapy in the future?’ (Fig 3), most of those surveyed indicated that telehealth could replace conventional therapy in adult speech and language disorder cases (82.6%), adult stuttering (79%), adult voice disorders (78%), pediatric speech disorders (73%), and pediatric stuttering (71%). The lowest percentages found in responses for cerebral palsy (18%) and autism (13%).

### Potential benefits and advantages of using telehealth

**Fig 4:**
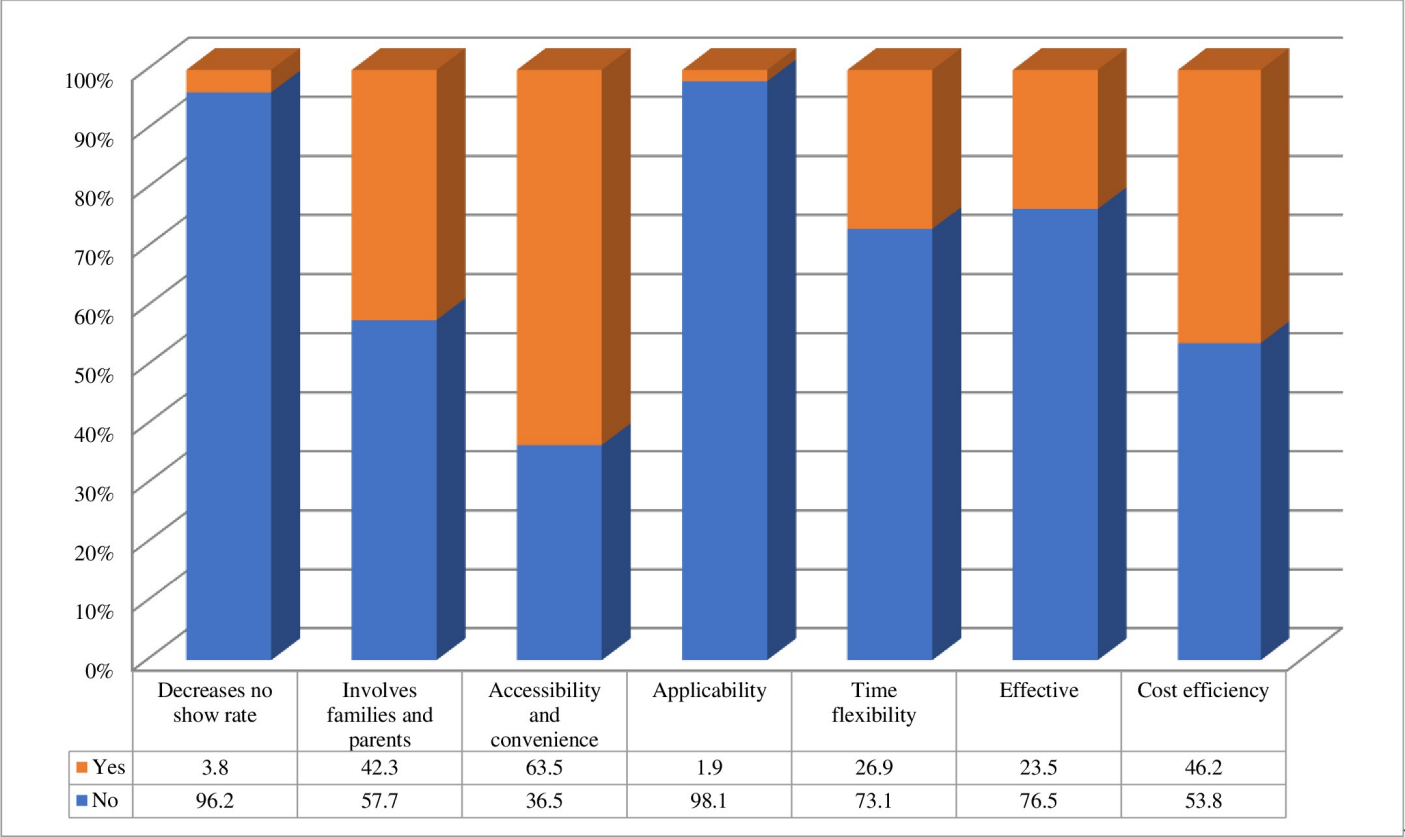
Benefits of telehealth practices (n = 61).

Part of our aim was to investigate the benefits of and barriers to using telehealth in the practice of SLPs; therefore, respondents who had used telehealth were invited to provide comments. The key themes arising from the responses were categorized as percentages based on the number of responses in each theme (Fig 4). The highest percentage of respondents reported that the use of telehealth is convenient and can be accessible (63.5%). Cost efficiency is another advantage of telehealth reported by the respondents who thought that this was especially true for patients living in rural cities (46%). A third advantage reported was the ability to engage patients with their parents or families in therapy sessions (42.3%). Regarding convenience, 27% of respondents reported that telehealth could be used at any time, and 24% of respondents viewed it as an effective solution for providing services and decreasing no show rates (4%) (Fig 4).

### Potential barriers and disadvantages of using telehealth

**Fig 5:**
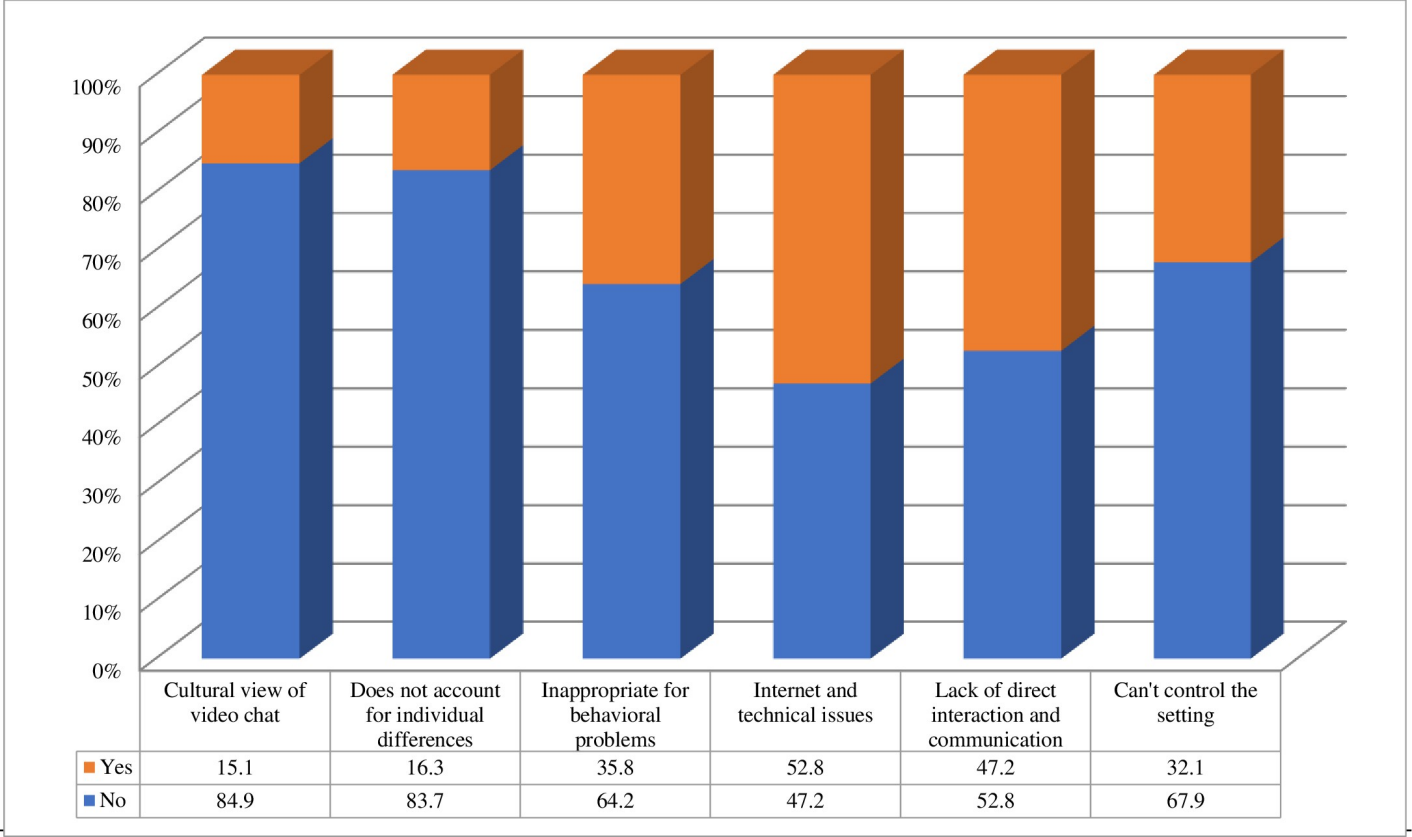
Barriers to telehealth practices (n = 61).

Other themes emerged from responses concerning the perceived barriers and disadvantages of using telehealth including internet and technical issues (53%), lack of direct interaction and communication (47%), the inability to address behavioral problems appropriately (36%), and difficulty controlling the therapeutic setting (32%) (Fig 5).

## Discussion

The findings provide insights into the experiences of SLPs and the emergence of telehealth before and during the outbreak.

### Professional practice during the COVID-19 pandemic

Almost all surveyed SLPs experienced changes in their service delivery. Many SLPs reported decreased work time and adopted new and different ways to deliver their services during the quarantine. Hence, a high proportion of SLPs provided different services remotely using a telephone for support and counseling (69%) and telehealth for assessment and therapy (55%).

### Use of telehealth by SLPs

The current study found that 67% of SLPs in Saudi Arabia have used telehealth services in their practice. Most survey respondents (80%) had started using telehealth for six months or less before the survey, whereas only 3% had used the service for more than two years. According to the findings of an earlier survey conducted by ASHA [14], over half of their respondents (57%) had used telehealth for more than three years, whereas only 17.9% had used it for less than one year. COVID-19 has motivated increased telehealth use: more than 90% of SLPs who have used telehealth for less than six months have started the service since the lockdown. Similar findings were reported in other countries [9, 22–24].

An encouraging finding of the survey is the intention of speech therapists to use telehealth in the future. About two-third of SLPs who either experienced (users) or did not experience (non-users) the use of telehealth responded positively to the idea of delivering their service via telehealth in the future (71% of users and 63% of non-users). The high level of interest surrounding telehealth among non-user SLPs was also reported in a study conducted by ASHA [13], wherein 47% of SLPs in the latter study who never use telehealth showed an interest in using it in the future. For SLPs who used telehealth during the pandemic, a study found that around half of the respondents showed interest in using telehealth in the future regardless of the pandemic [21].

Regarding the type of service or services provided using telehealth, the most common was counseling, followed by rehabilitation/treatment (69%), screening (63%), and evaluation/ assessment (56%); other services were conducted by less than half of the respondents. The high percentage of telehealth use for providing counseling and screening is consistent with ASHA’s findings [13]. Recent studies have reported evidence on the reliability of telehealth as a means of providing the assessment and screening of conditions that affect different areas, including articulation [25], aphasia [26], and dysphagia [27]. Notably, our findings regarding the use of telehealth to deliver other services, including treatment, contrasted with the findings reported by ASHA, but was consistent with Hill and Miller’s [28] report that the delivery of therapy was favored by 86% of their respondents.

The two most common kinds of cases in which respondents reported providing telehealth care were pediatric language (80%) and speech(70%) disorders, followed by stuttering (44.2%) and adult speech disorders (40%), which seem consistent with other findings reported in different studies [9, 14, 28, 29]. A small percentage of respondents used telehealth in other cases, and the telehealth was least frequently used in cases of voice disorders.

### Potential benefits of and barriers to using telehealth

Respondents listed a range of advantages and benefits to using telehealth in their practice, which was categorized into different themes, including convenience and accessibility, cost-efficiency, the involvement of families and parents, time flexibility, effectiveness, decrease in the no-show rate, and applicability. The identification of these benefits broadly supports the findings of other studies in this area. In terms of convenience and accessibility, telehealth can allow SLPs to offer their services to and expand in rural communities in order to provide services to those patients [21] [28, 30]. The last point is particularly important as most speech therapy services in Saudi Arabia are clustered in the largest cities; thus, patients need to travel long distances to reach services [31].

Accessibility and time efficiency were also considered strong motives for using telehealth [21, 32]. Following a study conducted by the Royal College of Speech & Language Therapists (RCSLT) [33] and the study conducted by Hao et al. [21], the involvement of families and parents was also seen by respondents as a positive point, because the work can also be carried out through caregivers. Furthermore, 46% of respondents considered telehealth to be a cost-effective method of treatment, which is consistent with Hill and Miller’s [28] Australian study. Although Hill and Miller’s participants reported that they viewed telehealth as cost-effective, they also demonstrated some concern about the expense of the technology. The same concern was raised by ASHA [13] and the Department of Health and Aging (DHA) [34] but did not appear in the current study. One possible explanation may be that 47% of respondents were working in governmental sectors at the time of the survey, and, therefore, expenses are covered by their institutions.

The findings regarding barriers to the use of telehealth were classified under different themes, including internet and technical issues, lack of direct interaction and communication, the ability to manage behavioral problems appropriately, and difficulty controlling the therapeutic setting. Some of these barriers seem to be consistent with the impressions reported in other research which found issues related to technology and clinical practice issues [22]. Different studies reported similar barriers and impracticalities, with some patients having disabilities that preclude the use of telehealth services and others preferring face-to-face contact [22, 24].

## Conclusion

The COVID-19 pandemic has caused dramatic changes in our lives in many different aspects, including clinical practice. The findings of this study reveal that the pandemic has significantly impacted the role of SLPs in Saudi Arabia and the way that services are delivered. In response to the pandemic, SLPs have been motivated to adopt telehealth at an accelerated pace; our results suggest that this will continue in the future. Despite concerns reported by the respondents, including technical and internet issues and difficulty using telehealth when behavioral problems are present, it appears that there is a general agreement that telehealth offers unique opportunities for improved care including easier access to services and a more cost-effective mode of therapy, especially for those living in rural areas. Furthermore, respondents suggested that there was a growing potential for the use of telehealth in areas such as counseling, rehabilitation/treatment, screening, and the evaluation of different cases, including speech and language disorders, stuttering, and voice disorders.

### Strength of the study

To our knowledge, this study is the first to investigate SLP services in Saudi Arabia and the changes adopted by SLPs as a response to the COVID-19 pandemic.

### Limitations and future directions

The survey has a limited number of participants, possibly because it only included SLPs who could be reached by e-mail or on social media platforms. Furthermore, the survey demonstrates the acceptance and accelerated usage of telehealth in SLP practices only for Saudi Arabia. Future studies should consider the use and efficiency of telehealth practices in different areas of speech and language therapy once the emergency measures related to the COVID-19 pandemic have been repealed.

## Supporting information

S1 File (SAV)
